# Strategic DAPT Interruption for Enlarging LV Pseudoaneurysm After Myocardial Infarction in a Dialysis Patient

**DOI:** 10.1016/j.jaccas.2026.107363

**Published:** 2026-03-16

**Authors:** Takeyoshi Kameyama, Yuki Kurose, Minoru Yambe, Takuya Shimizu, Shunsuke Kawamoto, Koji Kumagai

**Affiliations:** a Faculty of Medicine, Division of Cardiovascular Medicine, Tohoku Medical and Pharmaceutical University, Sendai, Japan; b Faculty of Medicine, Division of Cardiovascular Surgery, Tohoku Medical and Pharmaceutical University, Sendai, Japan

**Keywords:** left ventricle, myocardial infarction, percutaneous coronary intervention, stents, thrombus

## Abstract

**Background:**

Left ventricular pseudoaneurysm following acute myocardial infarction carries a high risk of rupture and typically requires surgical repair.

**Case Summary:**

A man in his 40s on maintenance hemodialysis presented with lateral-wall ST-segment elevation myocardial infarction during dialysis. Primary percutaneous coronary intervention successfully recanalized the occluded left circumflex artery. On day 26, echocardiography revealed a 15-mm left ventricular pseudoaneurysm that enlarged to 19 mm by day 38 (≈30% increase). Given prohibitive surgical risk due to multiple comorbidities and multidisciplinary heart team assessment, conservative management with strategic complete interruption of dual antiplatelet therapy was undertaken on day 39. Complete thrombosis occurred by day 53 without complications. Aspirin monotherapy was resumed on day 56. The 21-month follow-up demonstrated complete absorption.

**Discussion:**

Progressive enlargement rendered continued conservative observation insufficient. Following careful multidisciplinary evaluation in a surgically prohibitive, high-risk patient, strategic interruption of dual antiplatelet therapy was associated with complete thrombosis.

**Take-Home Messages:**

Small left ventricular pseudoaneurysms in high-risk patients may be managed conservatively with intensive surveillance. Rapid enlargement may signal impending rupture and, in selected cases with multidisciplinary consensus, warrant consideration of strategic dual antiplatelet therapy interruption.

## Visual Summary

**Figure:**
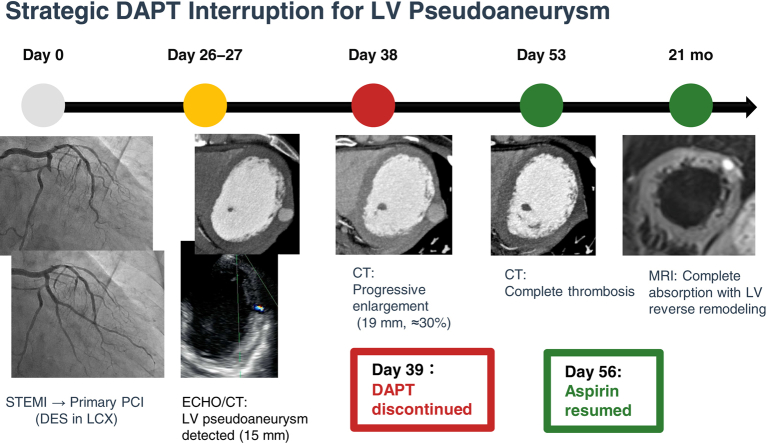

**Visual Summary:**
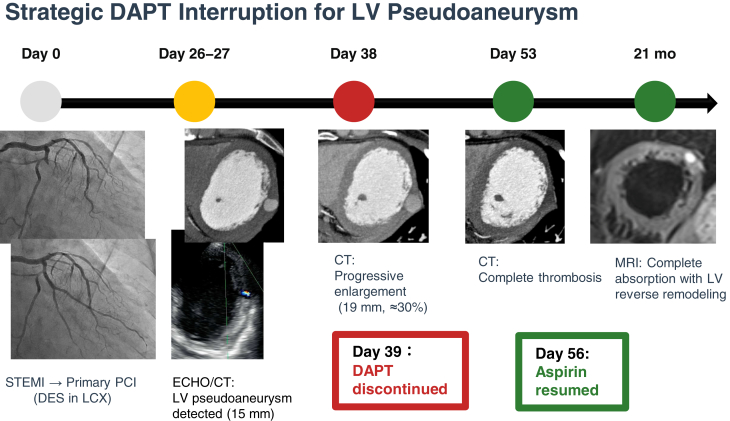
Timeline Illustrating the Clinical Course From Acute Myocardial Infarction and Primary PCI Through Pseudoaneurysm Detection, Enlargement, Strategic DAPT Interruption, and Eventual Thrombosis With Long-Term Resolution CT = computed tomography; DAPT = dual antiplatelet therapy; DES = drug-eluting stent; LCX = left circumflex artery; LV = left ventricle; MRI = magnetic resonance imaging; PCI = percutaneous coronary intervention; STEMI = ST-segment elevation myocardial infarction.

## History of Presentation

**Figure 1:**
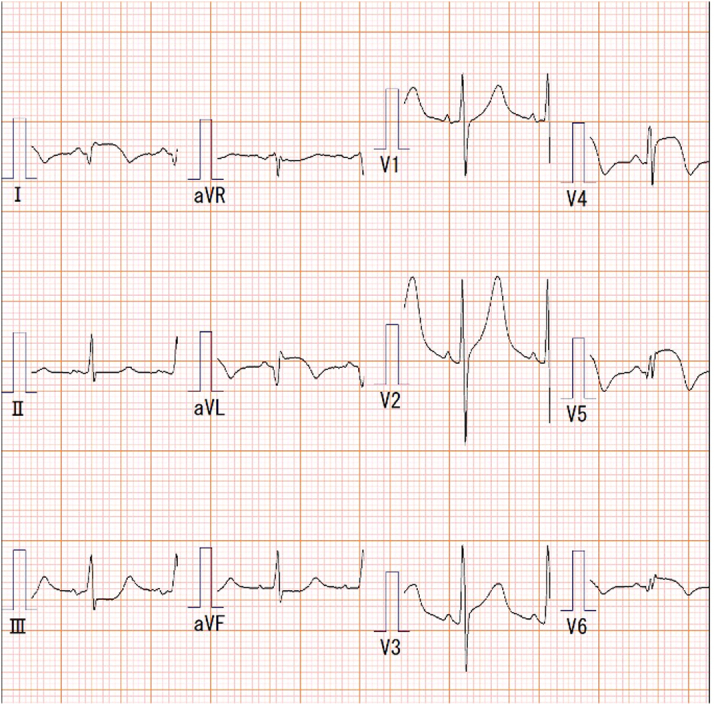
12-Lead Electrocardiogram on Presentation Showing ST-Segment Elevation in Leads I, aVL, and V_2_ to V_6_, Consistent With Acute Lateral-Wall ST-Segment Elevation Myocardial Infarction

A man in his 40s on maintenance hemodialysis was transferred from an outpatient dialysis facility due to fever and hypotension. He had developed chest pressure during the dialysis session. On arrival at our tertiary care center, physical examination revealed blood pressure of 100/79 mm Hg and a heart rate of 106/min. Electrocardiography showed ST-elevation in leads I, aVL, and V_2_ to V_6_, consistent with acute lateral-wall ST-segment elevation myocardial infarction (Figure 1). Laboratory findings included creatine kinase 1,805 U/L and troponin T >10.0 ng/mL.

**Take-Home Messages**

• Carefully selected high-risk patients with small left ventricular pseudoaneurysms may be managed conservatively with close imaging surveillance.
• In cases demonstrating rapid enlargement, strategic interruption of dual antiplatelet therapy may be considered following multidisciplinary consensus.

## Past Medical History

The patient had end-stage renal disease secondary to diabetic nephropathy requiring maintenance hemodialysis, type 2 diabetes mellitus with severe retinopathy resulting in near-complete vision loss, and hypertension. His father had myocardial infarction.

## Investigations

**Figure 2:**
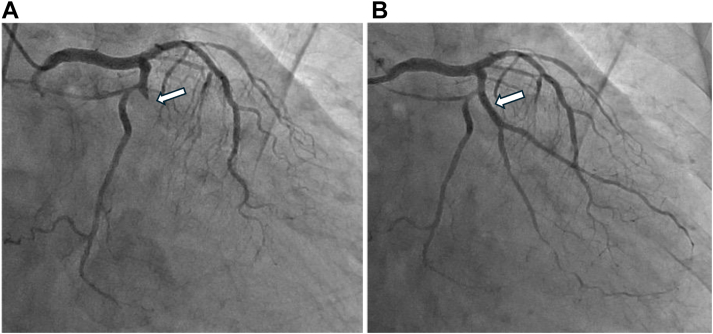
Coronary Angiography (A) Total occlusion of left circumflex artery at segment #12 before primary percutaneous coronary intervention. (B) Successful recanalization after drug-eluting stent implantation with final TIMI flow grade III. Arrows indicate the culprit lesion and the treated segment.

Emergency coronary angiography revealed total occlusion of the left circumflex artery at segment #12 (Figure 2A). Other coronary arteries were normal. Initial echocardiography showed lateral wall asynergy with a small pericardial effusion. Primary percutaneous coronary intervention was performed with a drug-eluting stent (Xience Skypoint 2.75 × 15 mm, Abbott Vascular), achieving TIMI 3 flow (Figure 2B).

Dual antiplatelet therapy (DAPT) with aspirin and prasugrel was initiated at the time of percutaneous coronary intervention.

The postprocedural course was complicated by persistent fever requiring intensive care unit management for 5 days.

## Differential Diagnosis

**Figure 3:**
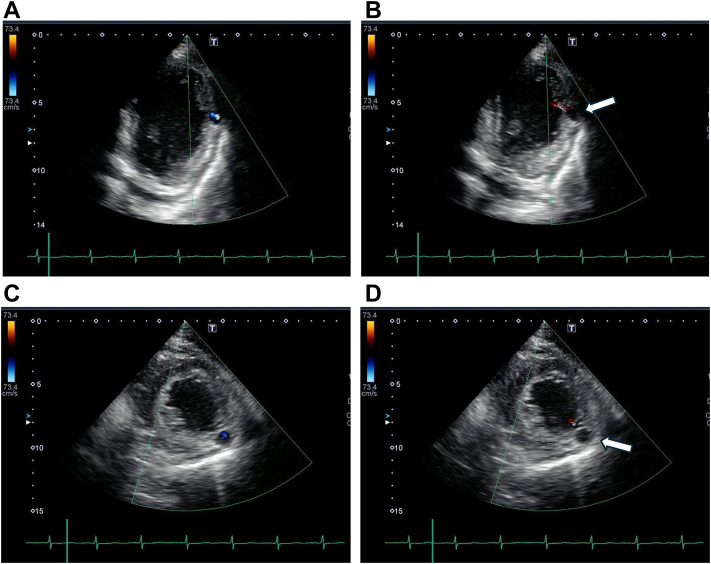
Echocardiography on Day 26 With Color Doppler Demonstrating Bidirectional Flow Communication (A) Long-axis view during systole. (B) Long-axis view during diastole. (C) Short-axis view during systole. (D) Short-axis view during diastole. Bidirectional flow through the narrow neck confirms communication between the left ventricle and pseudoaneurysm. Arrows in B and D indicate the pseudoaneurysm with flow communication.

On day 26, routine echocardiography revealed an echo-free space at the mid-lateral wall. Color Doppler imaging demonstrated bidirectional flow communication between the left ventricle and the cavity through a narrow neck, confirming active blood flow exchange during both systolic and diastolic phases (Figure 3, Videos 1 and 2). While initially reported as a diverticulum, the clinical context of recent transmural myocardial infarction raised suspicion for pseudoaneurysm.

The differential diagnosis included:

1. left ventricular pseudoaneurysm: suggested by a narrow neck, absence of myocardium in the cavity wall, bidirectional flow communication, and the clinical context of recent transmural MI;
2. left ventricular diverticulum: typically congenital, located at the cardiac apex, with preserved wall motion.

**Figure 4:**
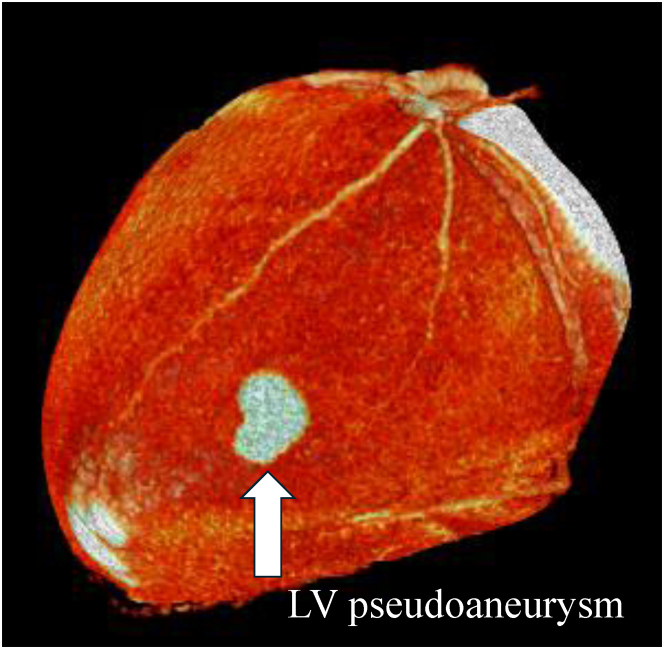
Three-Dimensional Contrast-Enhanced Computed Tomography Reconstruction Showing a 15-mm LV Pseudoaneurysm in the Lateral Wall (Arrow) LV = left ventricular.

However, diverticulum was excluded as serial echocardiography from days 0 to 5 showed no pre-existing structural abnormality. Contrast-enhanced computed tomography on day 27 demonstrated the absence of myocardial tissue within the cavity wall, confirming the diagnosis of pseudoaneurysm (Figure 4).

## Management

Following the diagnosis of a pseudoaneurysm, the multidisciplinary heart team considered 3 treatment options:

1. surgical patch repair requiring cardiopulmonary bypass,
2. percutaneous coil embolization as proposed by interventional radiology;
3. conservative management with strategic DAPT interruption (ie, complete interruption of both antiplatelet agents).

Given prohibitive surgical risk due to multiple comorbidities, recurrent infections, and severe visual impairment, the team elected to first attempt the least-invasive strategy of complete DAPT interruption on day 39, with plans to reconsider surgical or interventional options if this conservative approach failed. The surgical team further emphasized that continuation of DAPT, together with mandatory systemic heparinization during hemodialysis, would markedly increase perioperative bleeding risk, rendering immediate surgical patch repair extremely high risk. In the absence of prior reports, adopting complete DAPT interruption was extremely challenging, balancing rupture risk against stent thrombosis.

## Outcome and Follow-Up

**Figure 5:**
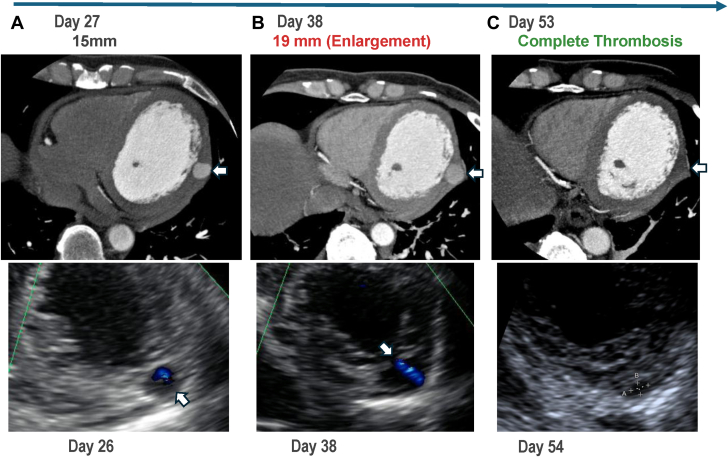
Serial Computed Tomography and Echocardiographic Imaging Demonstrating Left Ventricular Pseudoaneurysm Evolution Top row: Computed tomography images. Bottom row: echocardiography with color Doppler. (A) Day 26–27: pseudoaneurysm detected (15 mm on CT). (B) Day 38: enlargement (19 mm on CT). (C) Day 53–54: complete thrombosis. Arrows indicate the pseudoaneurysm on computed tomography and the corresponding echocardiographic findings.

Serial imaging confirmed complete thrombosis by day 53 without complications (Figure 5, Videos 3 and 4).

Aspirin monotherapy (single antiplatelet therapy) was resumed on day 56, and the patient remained hemodynamically stable throughout the observation period.

**Figure 6:**
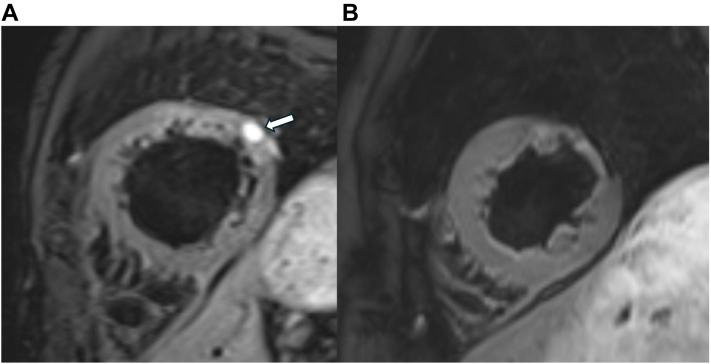
T2-Weighted Cardiac Magnetic Resonance Imaging (A) At 2 months: high signal intensity confirming organized thrombus. (B) At 21 months: complete resolution with left ventricular reverse remodeling. An arrow indicates the organized thrombus in A.

Cardiac magnetic resonance imaging at 2 months demonstrated organized thrombus (Figure 6A).

At 21-month follow-up, complete absorption with left ventricular reverse remodeling was observed (Figure 6B).

## Discussion

Left ventricular pseudoaneurysm is a rare but potentially life-threatening mechanical complication following acute myocardial infarction and has historically been associated with a high risk of rupture, often prompting surgical repair. Classic series have demonstrated that pseudoaneurysms with a narrow communication neck and small cavity size may exhibit relative stability and, in selected cases, allow for conservative management under close surveillance.^1^^,^^2^ Subsequent reports have further suggested that carefully selected patients without hemodynamic compromise may be managed nonsurgically with acceptable outcomes.^3^

In the present case, the pseudoaneurysm was initially small with a narrow neck and no evidence of hemodynamic instability, features previously associated with a lower immediate risk of rupture.^1^^,^^2^ However, progressive enlargement over a short interval raised concern for impending rupture. Serial multimodality imaging demonstrated preserved containment without evidence of free wall rupture or cardiac tamponade; nevertheless, these findings alone did not justify simple continuation of watchful waiting.

At this juncture, the multidisciplinary heart team formally considered all available management options, including surgical patch repair, percutaneous coil embolization, and conservative management with modification of antiplatelet therapy. Surgical patch repair, although definitive, was deemed prohibitively high risk because of multiple comorbidities, including maintenance hemodialysis and severe visual impairment. In particular, the surgical team emphasized that continuation of DAPT, together with mandatory systemic heparinization during hemodialysis, would markedly increase perioperative bleeding risk, rendering immediate surgical patch repair extremely high risk.^4^^,^^5^

Percutaneous treatment options, including transcatheter device closure and coil embolization, have been reported as alternatives in high-risk surgical patients.^6^ Coil embolization has been reported mainly in pseudoaneurysms of noninfarction etiology or postoperative settings, such as left ventricular outflow tract pseudoaneurysms.^7^ In the present case, given the favorable neck morphology, coil embolization was reserved as a potential rescue option should conservative management fail, while complete interruption of DAPT was initially attempted as the least invasive strategy.

A particularly challenging aspect of this case was the decision to completely interrupt DAPT shortly after drug-eluting stent implantation. In patients receiving maintenance hemodialysis, DAPT is associated with a substantially increased bleeding risk, highlighting the vulnerability of this population to hemorrhagic complications.^4^^,^^5^ Although brief interruption of both antiplatelet agents before a noncardiac surgery has been reported in selected hemodialysis patients with second-generation drug-eluting stent,^8^ conversely, interruption of DAPT—especially early after stent implantation—has been consistently associated with an increased risk of stent thrombosis, underscoring the inherent danger of this strategy.^9^^,^^10^ Notably, real-world registry data have demonstrated that the risk of stent thrombosis differs according to the mode of antiplatelet therapy interruption, with complete discontinuation of both agents conferring a significantly higher risk than continuation of single antiplatelet therapy.^10^

In this context, the morphological characteristics of the pseudoaneurysm were central to decision-making. The narrow communication neck and limited cavity size likely facilitated spontaneous thrombosis once antiplatelet therapy was withdrawn, consistent with pathophysiological mechanisms described in earlier reports of pseudoaneurysm stabilization.^1^^,^^2^ Importantly, interruption of DAPT was not undertaken as an endpoint but rather as a strategic, closely monitored intervention, adopted with the explicit understanding that continued enlargement or clinical deterioration would prompt reconsideration of surgical patch repair or percutaneous intervention once bleeding risk could be mitigated.

This case illustrates that, in carefully selected high-risk patients, strategic interruption of DAPT may be considered as a last-resort management option when rapid pseudoaneurysm enlargement raises concern for impending rupture and when surgical patch repair or percutaneous interventions are deemed prohibitively risky. While this approach should not be generalized, it underscores the importance of individualized, anatomy-driven decision-making supported by close follow-up and multidisciplinary expertise.

## Conclusions

Conservative management with intensive surveillance may be appropriate in carefully selected high-risk patients with small pseudoaneurysms, and strategic DAPT interruption may be considered as a management option in cases showing documented enlargement.

## Funding Support and Author Disclosures

The authors have reported that they have no relationships relevant to the contents of this paper to disclose.

## Appendix

Video 1 Transthoracic Echocardiography on Day 26 (Parasternal Short-Axis View) Demonstrating an Echo-Free Cavity at the Mid-Lateral Wall With Bidirectional Flow Communication Between the Left Ventricle and the Cavity Through a Narrow Neck on Color Doppler Imaging, Consistent With a Left Ventricular Pseudoaneurysm

Video 2 Transthoracic Echocardiography on Day 26 (Parasternal Long-Axis View) Demonstrating an Echo-Free Cavity Communicating With the Left Ventricle Through a Narrow Neck, Providing an Alternative View of the Left Ventricular Pseudoaneurysm

Video 3 Echocardiography on Day 38 (Short Axis View) With Color Doppler Demonstrating Continued Flow Communication Through the Pseudoaneurysm This video corresponds to the enlargement phase shown in Figure 5B, where the pseudoaneurysm had increased to 19 mm (≈30% enlargement from day 27).

Video 4 Echocardiography on Day 38 (Long Axis View) With Color Doppler Demonstrating Continued Flow Communication Through the Pseudoaneurysm This video provides an alternative view of the enlargement phase, corresponding to Figure 5B.
